# Development of ZD2767P–carboxypeptidase G2–ultrasound therapy against cisplatin-resistant cancer

**DOI:** 10.3389/fonc.2023.1151613

**Published:** 2023-05-18

**Authors:** Tinghe Yu, Xinya Li

**Affiliations:** Laboratory of Obstetrics and Gynecology, Second Affiliated Hospital, Chongqing Medical University, Chongqing, China

**Keywords:** ultrasound, prodrug-CPG2 therapy, cisplatin resistance, cancer therapy, ferroptosis

## 1 Introduction

Cancer chemotherapy is limited by adverse drug reactions (ADR) and chemoresistance. Prodrug–enzyme therapy is developed to improve the therapeutic outcome: an enzyme cleaves the prodrug to release the cytotoxic moiety. Of those enzymes tested in vivo, carboxypeptidase G2 (CPG2) is recommended for lacking human analogues; in the current regimen, CPG2 is linked to an antibody to realize targeting delivery, i.e., antibody directed enzyme prodrug therapy (ADEPT) (1). Consequently, prodrug activation is confined to tumors, improving the therapeutic precision. CMDA (4-[(2-chloroethyl) (2-mesyloxylethyl)amino]benzoyl-L-glutamic acid), or ZD2767P (4-[N,N-bis(2-iodoethyl)amino]phenoxycarbonyl-L-glutamic acid) combined with CPG2 has been clinically evaluated: main concerns are toxicities to noncancerous tissues and low anticancer efficacy, due to off-target activation of prodrugs and the leakage of activated drugs from tumors into circulation (2).

## 2 Limitations of prodrug–CPG2 therapy noted in clinical trials

ADEPT has been clinically tested on cancers expressing carcinoembryonic antigen (CEA), and major limitations are described.

### 2.1 Low targeting efficacy

Intravenously infusing CPG2 linked to a F(ab)_2_ fragment of mouse anti-CEA antibody (3000 U/m^2^) led to a median CPG2 level in tumors of 0.010 U/g (0–0.208) on prodrug day (2–9 day), the median serum level was 0.037 U/mL (0.011–0.180), and the median level ratio of tumor to blood was 0.4 (0–10.4) (3). The low targeting efficacy plays an important part in low anticancer efficacy and ADR.

### 2.2 Immunogenicity

Both CPG2 and the linked antibody can induce production of anti-drug antibody (ADA). CPG2 conjugated to a mouse anti-CEA F(ab)_2_ antibody caused anti-mouse IgG antibody in 26/26 cases, and anti-CPG2 antibody in 25/26 cases (3). ADA may affect the pharmacokinetic (PK) behavior, and certain ADA is the neutralizing antibody (NAb) that can inhibit activity of CPG2. ADA may hinder repeated cycles.

### 2.3 Specific activity and PK concerns

Specific activities of CPG2, CPG2 linked to anti-CEA single-chain Fv (scFv) antibody, and CPG2 linked to anti-CEA F(ab)_2_ antibody were 437, 128 and 80 U/mg, with molecular masses of about 83, 143 and 183 kDa, respectively (4–8). A higher dose of conjugate is therefore needed to realize an equivalent CPG2 dose. The nonproportional decrease in specific activity suggests conformational changes that may impact PK.

PK of conjugated CPG2 differed from that of CPG2 (**Table S1**) (3–6, 9). PK of CPG2 was proportional, but that of conjugated CPG2 remained unclear (9). PK proportionality of ZD2767P was not validated (3, 5). Intratumoral and intracellular PK of CPG2, prodrug and activated drug have not been elucidated yet.

## 3 Development of ZD2767P–CPG2–US therapy

Ultrasound (US) can modulate the CPG2 activity, being a means to adjust the output rate of activated drug in prodrug–CPG2 therapy (10). Further, ultrasound permeabilizes vessels and cellular membranes, favoring the influx of drugs into cells to enhance the drugs’ action (i.e., sonochemotherapy) (11–13). ZD2767P-CPG2-US therapy is designed and tested on cisplatin-resistant cancer, considering that chemoresistance has been a clinical challenge (14).

### 3.1 Anticancer efficacy in vitro and in vivo

Human ovarian cancer cells SKOV3 and SKOV3/DDP, and lung cancer cells A549 and A549/DDP were used; SKOV3/DDP and A549/DDP were cisplatin-resistant sublines. Cells received ZD2767P+CPG2 or ZD2767P+CPG2+US. ZD2767P+CPG2+US led to a lower cell-survival percentage and a higher apoptosis percentage compared with ZD2767P+CPG2, with a lower IC_50_ (half maximal inhibitory concentration) of ZD2767P (**Figure S1**). The interaction analysis utilizing cell-death percentages indicated that ultrasound synergized ZD2767P+CPG2. Noticeably, resistant and sensitive cells had similar responses to ZD2767P+CPG2+US (15, 16).

The cytotoxic molecule of the ZD2767P+CPG2 regimen is ZD2767D (4-[N,N-bis(2-iodoethyl)amino]phenol) that attacks DNA (17). DNA damage was analyzed, where the alkaline comet assay detected both single- (SSB) and double-strand break (DSB) and the neutral assay detected DSB. ZD2767P+CPG2+US led to a higher comet percentage, and the neutral-comet percentage approached to the alkaline- one, indicating that DSB was the leading mode of DNA damage (**Figure S1**). Directly inducing DSB was one of the reasons that ZD2767P+CPG2+US efficiently deactivated resistant cells. Cisplatin primarily causes SSB, most DSB is evolution of SSB, and unrepairable DSB triggers apoptosis. Resistant cells have apoptosis malfunction and a higher capacity of DNA repair (14). Thus, the apoptosis pathway of ZD2767P+CPG2+US differed from that of cisplatin, which should be elucidated.

ZD2767P-CPG2-US therapy was tested in subcutaneous tumors. Volume-inhibitory rates in groups ZD2767P+CPG2+US and ZD2767P+CPG2, were 26.5% and 20.4% in SKOV3 tumors, 81.6% and 36.8% in SKOV3/DDP tumors, 63.5% and 39.7% in A549 tumors, and 70.1% and 50.0% in A549/DDP tumors, respectively. No serum active CPG2 and no severe ADR were noted (15, 16). The survival benefit was assessed in nude mice bearing advanced orthotopic ovarian tumors. In animals bearing SKOV3 or SKOV3/DDP tumors, ZD2767P+CPG2 or ZD2767P+CPG2+US treatments prolonged the survival time from 25.0 ± 1.6 to 33.0 ± 3.5 or 39.2 ± 1.8 days, or from 8.7 ± 3.9 to 16.2 ± 4.8 or 22.3 ± 7.3 days, respectively (15).

### 3.2 Intracellular PK of ZD2767D

The intracellular level of ZD2767D was assayed. The peak level (C_max_), area under the drug level vs. time curve from zero to last measurable time point (AUC_last_), and mean residence time from zero to last measurable level (MRT_last_) in group ZD2767P+CPG2+US were higher than those in group ZD2767P+CPG2, but with a lower volume of distribution (V_z_) (**Table S2**). C_max_ and AUC determine the efficacy of a drug. Therefore, insonation altered PK of ZD2767D to enhance the action of ZD2767P+CPG2.

Percentages of cell-uptake were 4.3–4.5% and 7.9–8.7%, in groups ZD2767P+CPG2 and ZD2767P+CPG2+US respectively, indicating that ZD2767D entered cells *via* passive diffusion and that ultrasound increased the drug influx. Ultrasonic cavitation improved the CPG2 activity to increase the ZD2767D level in the extracellular media during a period of time (10, 15). Cavitation permeabilized cellular membranes, enhancing the transmembrane influx of drugs and prolonging the duration of drug influx. These effects increased the intracellular amount of ZD2767D. The low anticancer efficacy of ZD2767P+CPG2 in humans is partly due to a short t_1/2_ of ZD2767D in blood (≈2 min) (17). MRT_last_ was 14–29 min in cells. Hence, transferring extracellular ZD2767D into cells can prolong the retention time in vivo.

t_1/2_, AUC from zero to infinity (AUC_inf_), and MRT from zero to infinity (MRT_inf_) had greater standard deviations, being inappropriate parameters to assess PK (**Table S2**). These parameters utilized extrapolated values. Heterogeneity of cancer cells and the experimental manner (a different cell population was adopted at each time point in a cell PK trial) led to drastic variance between individuals.

PK proportionality that depended on cell type, drug and the behavior of ultrasound, was confirmed only in A549 and A549/DDP cells in the ZD2767P+CPG2 regimen (15, 16). Thus, nonproportional PK should be considered when planning a therapy.

### 3.3 Cell-death modes

Cell-death modes were explored in A549 and A549/DDP cells. The cell-survival percentage was increased when inhibiting apoptosis with Z-VAD-fmk or inhibiting ferroptosis with ferrostatin-1, but was not changed when inhibiting autophagy with 3-methyladenine; the highest cell-survival percentage occurred when using both Z-VAD-fmk and ferrostatin-1 (16). These data indicated that either ZD2767P+CPG2 or ZD2767P+CPG2+US deactivated cells *via* apoptosis and ferroptosis pathways.

Accumulation of iron and reactive oxygen species (ROS), and a decrease in glutathione peroxidase 4 (GPX4) determine ferroptosis. Transferrin (TF) imports irons and ferroportin (FPN) exports irons (18). The expression level of TF was increased but that of FPN was decreased, being consistent with a higher intracellular level of iron. ZD2767P+CPG2+US treatments led to a higher ROS level, and the GPX4 level was decreased (16). GPX4 can counteract lipid peroxidation, and glutathione (GSH) is a cofactor (19). Ultrasound reduced the GSH level in cisplatin-resistant ovarian cancer cells, which can decrease the activity of GPX4 (20, 21). A decrease in amount and/or activity of GPX4 favors lipid peroxidation (19). Thus, ferroptosis played a part in cell death after ZD2767P+CPG2+US treatments.

DNA damage may be the primary initiator for both apoptosis and ferroptosis. Signals of DNA damage were transmitted to mitochondria to trigger apoptosis (22). Ferroptosis was an alternative cell-death mode for unrepairable DNA damage (23).

Ferroptosis was verified in A549 and A549/DDP subcutaneous tumors. Compared with tumors receiving ZD2767P+CPG2 treatments, a higher apoptosis level and a lower GPX4 level were noted in tumors receiving ZD2767P+CPG2+US treatments, demonstrating that in vivo anticancer action was due to apoptosis and ferroptosis (**Figure S2**) (16).

**Figure 1:**
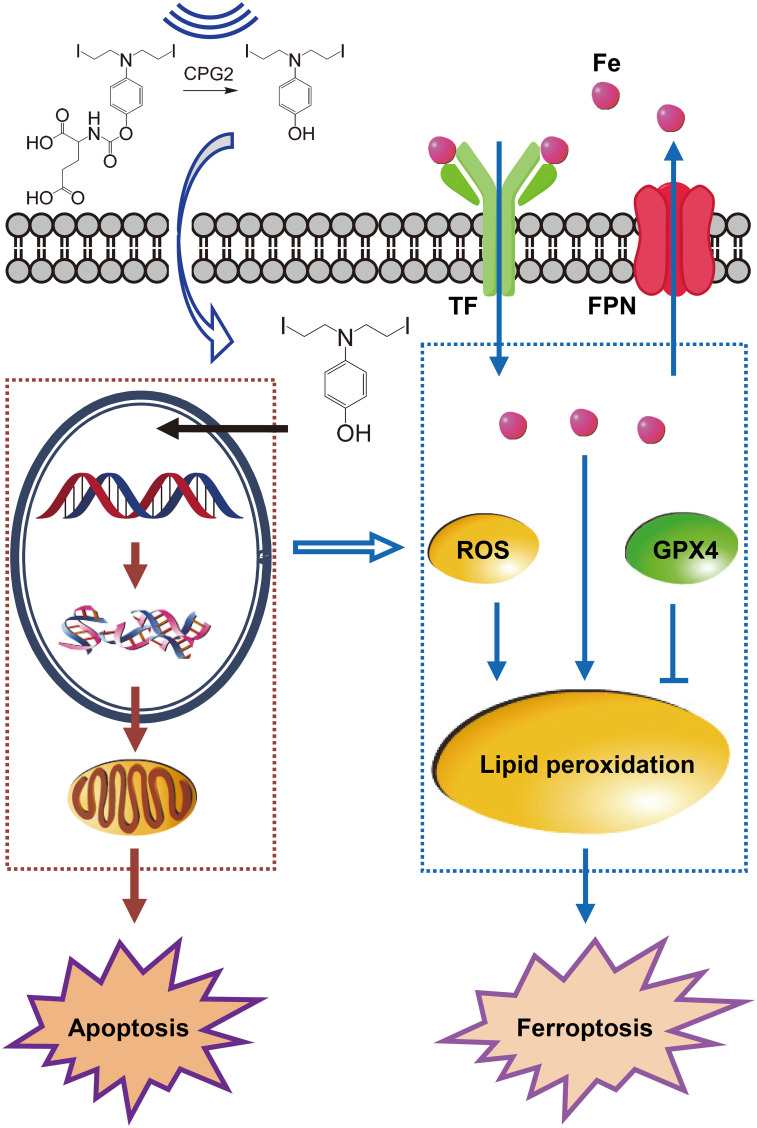
Pharmacokinetics–pharmacodynamics in ZD2767P-CPG2-US therapy. Insonation improved the CPG2 activity, and impelled ZD2767D molecules to enter cells; higher C_max_ and AUC_last_ of ZD2767D created severer DNA damage to trigger apoptosis. Up-regulation of transferrin (TF) and down- of ferroportin (FPN) led to iron accumulation; the level of reactive oxygen species (ROS) was increased, and that of glutathione peroxidase 4 (GPX4) was decreased; these effects caused lipid peroxidation, and eventually resulted in ferroptosis. Thus, ZD2767P+CPG2+US deactivated calls *via* apoptosis and ferroptosis pathways.

Thus, PK–PD (pharmacodynamics) of ZD2767P-CPG2-US therapy was: insolation led to a higher intracellular level of ZD2767D with a longer retention time, which created severer DNA damage; unresolved DSB triggered apoptosis and ferroptosis (**Figure 1**). Ferroptosis offset the apoptosis deficiency, thereby deactivating more cells to improve the anticancer efficacy.

## 4 Implications for future development

### 4.1 Repeated cycles

Immunogenicity of CPG2 and linked antibodies hinders repeated cycles (2). In mice bearing orthotopic ovarian tumors, up to 3 times of treatments were conducted, indicating that repeated cycles were feasible (15). When using CPG2 to rescue methotrexate intoxication, that immunogenicity of CPG2 affected PK/PD of CPG2 was not observed. Reasons may be: cancer patients were in the immunosuppressed status; the catalytic domain of carboxypeptidases was highly conserved among species, so human had partial immune tolerance to CPG2; CPG2 had a short t_1/2_ (24). Only NAb can inhibit the CPG2 activity, and therefore ADA to CPG2 may not be a crucial factor for repeated treatments. ADA to linked antibodies can hamper gathering of the CPG2–antibody conjugate to tumors, decreasing the intratumoral CPG2 level and the CPG2 level ratio of tumor to normal tissues. Distinct implications of ADA to CPG2 and to linked antibodies need particular concerns.

PK of prodrug and/or activated drug is a window to understanding the impact of ADA on prodrug–CPG2 therapy. Repeated treatments can be administrated if PK has slight changes. However, above moderate PK changes should be a contraindication for the next cycle.

### 4.2 Ferroptosis

Ferroptosis in ZD2767P-CPG2-US therapy suggested a strategy against resistant cancers, since inducing nonapoptotic cell death can combat chemoresistance (25). A cancer is heterogeneous with multiple cell subpopulations with distinct features (26–28). Ferroptosis bypasses the apoptosis pathway to lead to cell death in certain subpopulations, and offsets the apoptosis insufficiency in other subpopulations, thereby deactivating abundant cancer cells.

Ferroptosis mechanisms/pathways (including metabolic changes involved) in ZD2767P-CPG2-US therapy, and its weight in cell death should be elucidated (29). Whether other cell-death modes (e.g., necroptosis) play a part is the other concern; if so, their interactions with apoptosis or ferroptosis should be addressed.

### 4.3 Therapeutic regimen

In clinical ZD2767P–CPG2 therapy, CPG2–antibody conjugate was intravenously infused, and ZD2767P was intravenously injected when the serum CPG2 level fell below the preset threshold. The interval drastically varied between cases: 2–9 days for CPG2–F(ab)_2_ antibody and 5–37.2 h for CPG2–scFv antibody (3, 5). Indeed, a low serum CPG2 level unnecessarily indicates an adequate amount of CPG2 in tumors and a high level ratio of tumor to normal tissues.

In preclinical trials, ZD2767P was intravenously injected, followed by intratumoral injection of CPG2 after 10 min, and then the tumor received insonation after 5 min (15, 16). A small volume of injecting CPG2 reduced diffusion into blood and realized a high intratumoral level, favoring production and confinement of ZD2767D in the tumor. The interval between CPG2 and insonation was set to make all ZD2767P molecules in the tumor hydrolyzed: time required (min) = amount of prodrug (mol)/[amount of CPG2 (U) × catalytic rate (mol min^-1^ U^-1^)] (15). More ZD2727D molecules entered in cancer cells under insonation to increase C_max_ and AUC_last_, improving the anticancer efficacy.

Intracellular ZD2767P is a deadweight loss of prodrugs, since CPG2 cannot cross cellular membranes (15). Given better safety and efficacy, intratumorally injecting CPG2 can be used, which saves antibodies and omits the in vivo travel process. This manner can be first tested on shallowly located tumors. Administration regimens of CPG2 and ZD2767P should be explored in following trials.

### 4.4 Therapeutic precision

Therapeutic precision is mainly determined by the absolute and relative amount of CPG2 in tumors, which depends on the affinity and specificity of linked antibodies in ADEPT. Many in vivo factors can interfere the linked antibody and CPG2; CPG2 may affect function of the linked antibody and vice versa. These factors impact the travel of CPG2–antibody conjugate to the tumor and the conjugate’s binding to cancer cells, decreasing the therapeutic precision.

Injecting CPG2 and insonating the tumor can be guided and monitored by imaging. Ultrasonically enhanced transfer of ZD2767D into cancer cells prolongs the in vivo retention time and decreases its leakage into blood. Therefore, ZD2767P–CPG2–US therapy is with high therapeutic precision.

### 4.5 Insonation

Both CPG2 modulation and membrane permeabilization mainly ascribed to ultrasonic cavitation. The cavitation level calibrated by the yield of free radicals did not proportionally increase with prolonging insonation time (10). Increases in membrane permeabilization and in drug influx are non-linear (11, 12). The cavitation field that determines the therapeutic field is inhomogeneous (15, 30). Cavitation generates free radicals that increase the ROS yield, and ROS regulates both apoptosis and ferroptosis (21, 22). Techniques that quantify in vivo free radicals due to cavitation therefore should be developed.

Focused ultrasound was adopted in preclinical trials, which efficiently delivered ultrasonic energy to the tumor (15, 16). Consequently, increases in the activity of CPG2, membrane permeabilization and ZD276D influx were confined to the tumor, resulting in higher safety and efficacy. Thus, focused ultrasound is preferred for ZD2767P–CPG2–US therapy.

In summary, ZD2767P+CPG2+US deactivates cancer cells *via* ultrasonically modulating CPG2 and permeabilizing cellular membranes. PK–PD is that higher C_max_ and AUC_last_ of ZD2767D directly caused DSB, resulting in apoptosis and ferroptosis. ZD2767P-CPG2-US therapy can address the low targeting and intracellular PK issues of ZD2767P–CPG2 therapy, and can realize a precision therapy against chemoresistant cancers. Cancer types/subtypes that are indications for this therapy should be determined in following trials.

## Author contributions

All authors listed have made a substantial, direct, and intellectual contribution to the work and approved it for publication.
