# Supplementary material for: Development of ZD2767P–carboxypeptidase G2–ultrasound therapy against cisplatin-resistant cancer
Source: Front Oncol. 2023 May 18;13:1151613. doi: 10.3389/fonc.2023.1151613 (PMC10233003; doi:10.3389/fonc.2023.1151613)
Supplement: Supplementary file 1 [file DataSheet_1.docx]

**Table S1** Clinical PK of CPG2 and prodrug ZD2767P

|  | CPG2‒F(ab)_2_ | CPG2‒scFv | CPG2 (50 U/kg) | | |
| --- | --- | --- | --- | --- | --- |
|  |  |  | Westerner | Chinese | Japanese |
| CPG2 |  |  |  |  |  |
| C_max_ (μg/mL) |  |  | 3.1±0.8  (healthy)  2.4‒2.5  (cancer) | 2.3±0.4  (healthy)  3.8±0.7  (cancer) | 2.4±0.3  (healthy) |
| t_1/2_ (h) | 8.95‒15.49  (3000 U/m^2^) | 1.96  (3000 U/m^2^)  4.6  (5000 U/m^2^) | 9.0±3.2  (healthy)  3.3‒3.6  (cancer) | 7.2±0.7  (healthy)  4.8±1.0  (cancer) | 7.2±0.4  (healthy) |
| AUC_inf_ (μg.h/mL) |  |  | 23.4±6.8  (healthy)  12.8‒13.3  (cancer) | 17.6±5.0  (healthy)  19.3±10.5  (cancer) | 20.9±3.2  (healthy) |
| ZD2767P |  |  |  |  |  |
| Dosage (mg/m^2^) | 4.9‒18.63 | 12.42‒537.6 |  |  |  |
| C_max_/C_0_ (μg/mL) | 1.1‒5.2 (C_max_) | 4.2‒235 (C_0_) |  |  |  |
| t_1/2_ (min) | 4.7‒14.5 | 7.0‒14.4 |  |  |  |
| AUC (μg.min/mL) | 8.0‒61 | 34‒3408 (AUC_inf_) |  |  |  |

C_max_: maximum concentration; C_0_: concentration at 0 min; t_1/2_: half-life; AUC: area under the concentration vs. time curve; AUC_inf_: AUC from zero to infinity.

F(ab)_2_ and scFv were anti-CEA antibody.

References

Francis RJ, Sharma SK, Springer C, et al., A phase I trial of antibody directed enzyme prodrug therapy (ADEPT) in patients with advanced colorectal carcinoma or other CEA producing tumours. Br J Cancer 2002, 87: 600-607.

Mayer A, Francis RJ, Sharma SK, et al., A phase I study of single administration of antibody-directed enzyme prodrug therapy with the recombinant anti-carcinoembryonic antigen antibody-enzyme fusion protein MFECP1 and a bis-iodo phenol mustard prodrug. Clin Cancer Res 2006, 12: 6509-6516.

Phillips M, Smith W, Balan G, et al., Pharmacokinetics of glucarpidase in subjects with normal and impaired renal function. J Clin Pharmacol 2008, 48: 279-284.

Fukaya Y, Kimura T, Yoshimura K, et al., A dose-confirmation phase 1 study to evaluate the safety and pharmacology of glucarpidase in healthy volunteers. Clin Pharmacol Drug Dev 2022, 11: 364-371.

**Table S2** Intracellular PK of ZD2767D at 100μM ZD2767P

|  | Ovarian cancer cell | | | | Lung cancer cell | | | |
| --- | --- | --- | --- | --- | --- | --- | --- | --- |
|  | SKOV3 | | SKOV3/DDP | | A549 | | A549/DDP | |
|  | ZD2767P+CPG2 | ZD2767P+CPG2+US | ZD2767P+CPG2 | ZD2767P+CPG2+US | ZD2767P+CPG2 | ZD2767P+CPG2+US | ZD2767P+CPG2 | ZD2767P+CPG2+US |
| C_max_ (nmol/mg) | 3.63±0.23 | 6.88±0.51^a^ | 4.12±0.43 | 8.06±0.40^a^ | 3.89±0.13 | 7.42±0.93^a^ | 3.96±0.23 | 7.83±1.12^a^ |
| t_1/2_ (min) | 57.66±31.00 | 95.47±25.54 | 81.85±15.08 | 289.35±145.46 | 131.18±67.42 | 522.72±499.00 | 113.47±71.52 | 433.48±185.49 |
| AUC_last_ ((nmol/mg) min) | 91.06±11.53 | 298.38±14.27^a^ | 103.11±9.00 | 366.86±10.13^a^ | 101.91±4.78 | 343.62±29.46^a^ | 104.12±5.27 | 369.00±33.49^a^ |
| AUC_inf_ ((nmol/mg) min) | 322.85±167.77 | 849.34±217.28^a^ | 486.05±32.24 | 2759.49±1224.42 | 749.45±398.48 | 4234.06±3623.94 | 649.08±367.48 | 3884.71±1422.63 |
| MRT_last_ (min) | 14.31±0.98 | 27.18±1.15^a^ | 14.86±0.13 | 28.76±0.20^a^ | 15.01±0.27 | 28.67±0.29^a^ | 14.91±0.32 | 28.86±0.42^a^ |
| MRT_inf_ (min) | 84.18±45.23 | 137.47±38.57 | 120.09±21.72 | 418.28±207.06 | 190.96±97.62 | 752.93±719.37 | 165.03±103.03 | 624.22±267.57 |
| V_z_ (μM/(nmol/mg)) | 25.52±1.27 | 16.18±1.16^a^ | 24.17±2.77 | 14.81±1.09^a^ | 25.37±0.58 | 16.80±2.16^a^ | 24.71±1.63 | 15.76±1.88^a^ |
| Cl (μM/(nmol/mg)/min) | 0.42±0.31 | 0.12±0.03 | 0.21±0.01 | 0.04±0.02^a^ | 0.16±0.08 | 0.04±0.02 | 0.19±0.10 | 0.03±0.01 |

C_max_: peak level; t_1/2_: half-life; AUC_last_: area under the drug level vs. time curve from zero to last measurable level; AUC_inf_: AUC from zero to infinity; MRT_last_: mean residence time from zero to last measurable level; MRT_inf_: MRT from zero to infinity; V_z_: volume of distribution; Cl: clearance.

Data were from 3 independent trials. a: vs. without US, p < 0.05.

C_max_, AUC_last_ and MRT_last_ were from Liu et al..

References

Liu Q, Zhong X, Zhang Y, et al., Ultrasound enhances ZD2767P‒carboxypeptidase G2 against chemoresistant ovarian cancer cells by altering the intracellular pharmacokinetics of ZD2767D. Mol Pharm 2020, 17: 1922-1932.

Liu Q, Li X, Luo Y, et al., Ultrasonically enhanced ZD2767P‒carboxypeptidase G2 deactivates cisplatin-resistant human lung cancer cells. Oxid Med Cell Longev 2022, 2022: 9191233.

**
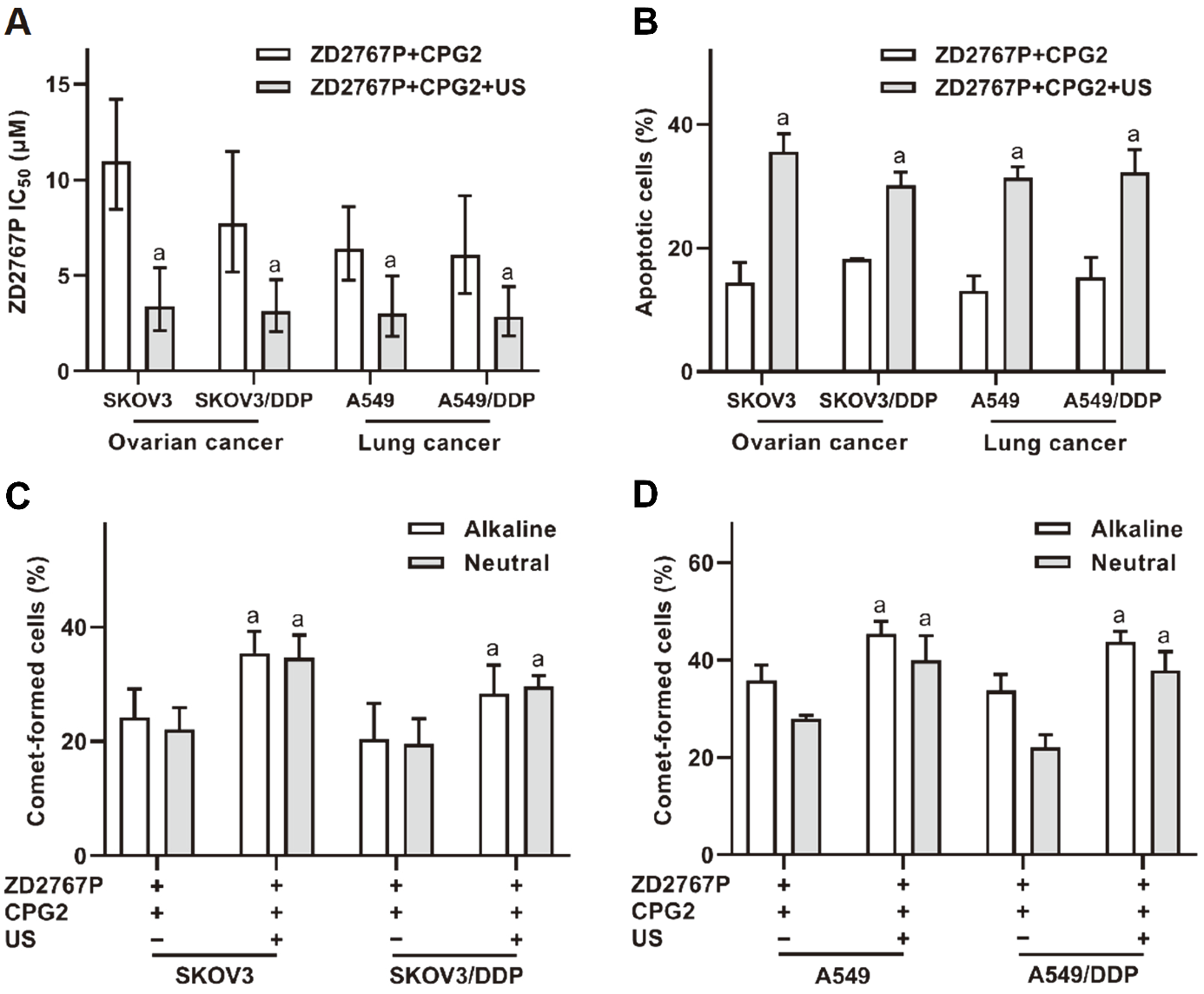
**

**Figure S1** Cells’ responses to ZD2767P+CPG2 or ZD2767P+CPG2+US. (**A**) IC_50_ of ZD2767P: a lower value was noted in cells exposed to ZD2767P+CPG2+US. (**B**) Apoptotic percentages: more apoptosis cells were detected after exposed to ZD2767P+CPG2+US. (**C, D**) DNA damage detected with the comet assays: a higher comet percentage was noted in cells exposed to ZD2767P+CPG2+US; the neutral-comet percentage approached to the alkaline- one, indicating that double stand break mainly ascribed to direct damage. a: vs. ZD2767P+CPG2, p<0.05. Data were from Liu et al..

References

Liu Q, Zhong X, Zhang Y, et al., Ultrasound enhances ZD2767P‒carboxypeptidase G2 against chemoresistant ovarian cancer cells by altering the intracellular pharmacokinetics of ZD2767D. Mol Pharm 2020, 17: 1922-1932.

Liu Q, Li X, Luo Y, et al., Ultrasonically enhanced ZD2767P‒carboxypeptidase G2 deactivates cisplatin-resistant human lung cancer cells. Oxid Med Cell Longev 2022, 2022: 9191233.

**
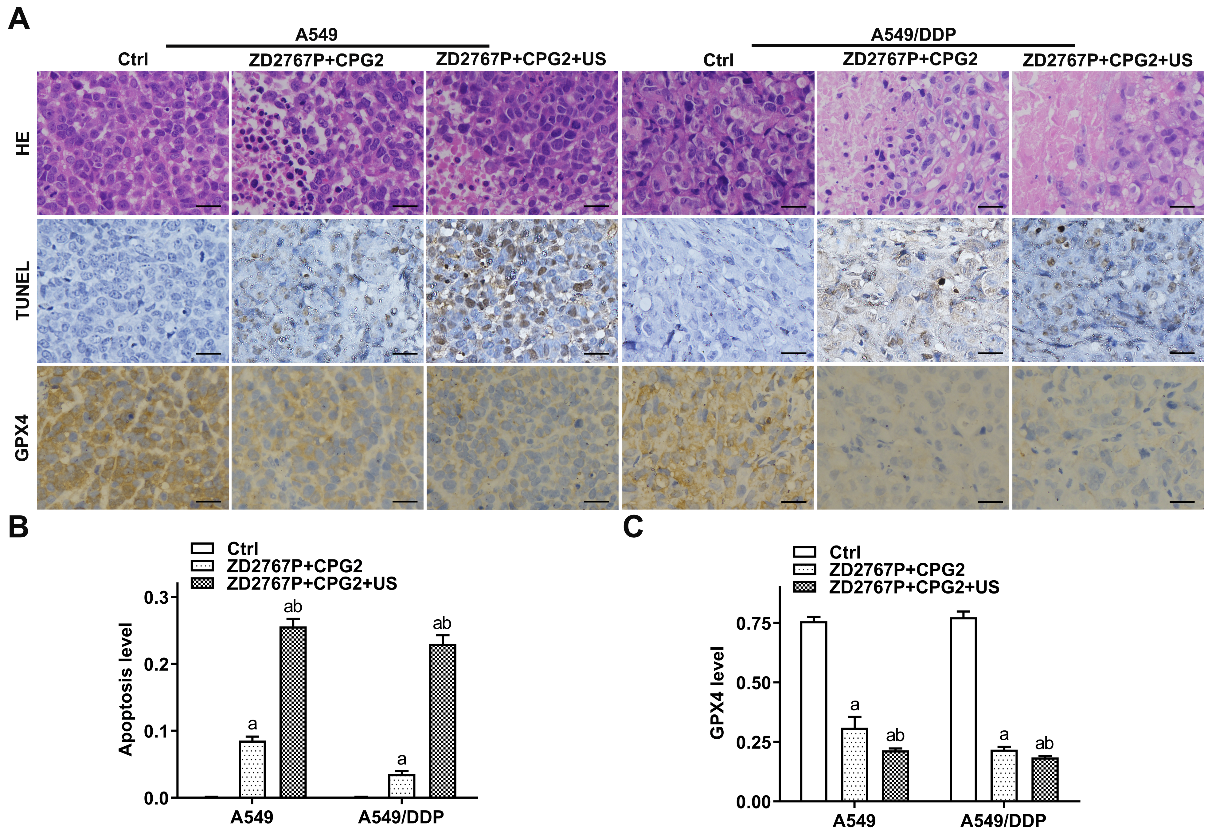
**

**Figure S2** Apoptosis (TUNEL) and glutathione peroxidase 4 (GPX4) in A549 and A549/DDP tumors. A higher apoptosis level and a lower GPX4 level were detected in tumors receiving ZD2767P+CPG2+US treatments in comparison with tumors receiving ZD2767P+CPG2 treatments, demonstrating occurrence of apoptosis and ferroptosis. a: vs. Ctrl, p < 0.05; b: vs. ZD2767P+CPG2, p < 0.05. Data were from Liu et al..

References

Liu Q, Li X, Luo Y, et al., Ultrasonically enhanced ZD2767P‒carboxypeptidase G2 deactivates cisplatin-resistant human lung cancer cells. Oxid Med Cell Longev 2022, 2022: 9191233.
